# Supplementary material for: End Sequence Analysis Toolkit (ESAT) expands the extractable information from single-cell RNA-seq data
Source: Genome Res. 2016 Oct;26(10):1397–410. doi: 10.1101/gr.207902.116 (PMC5052061; doi:10.1101/gr.207902.116)
Supplement: Supplemental Material [file supp_gr.207902.116_Supp_Materials.tar.gz › ESAT-esat_v0.1/doc/html/dir_25a1901adf9b1f69893c2ccd3af2349f.html]

ESAT: java/umms/core/model/score Directory Reference


ESAT


- Main Page
- Related Pages
- Packages
- Classes
- Files

All Classes Namespaces Files Functions Variables Enumerator Pages

- java
- umms
- core
- model
- score

score Directory Reference

Directory dependency graph for score:

|  |  |
| --- | --- |
| Files | |
| file | AbstractListScore.java |
|  | |
| file | BasicScanStatisticScore.java |
|  | |
| file | BinomialEnrichmentScore.java |
|  | |
| file | CountScore.java |
|  | |
| file | CoverageScore.java |
|  | |
| file | EndInWindowCountScore.java |
|  | |
| file | LengthScore.java |
|  | |
| file | MultiScore.java |
|  | |
| file | NewRatioScore.java |
|  | |
| file | PoissonEnrichmentScore.java |
|  | |
| file | RatioScore.java |
|  | |
| file | ScanStatisticScore.java |
|  | |
| file | StartInWindowCountScore.java |
|  | |
| file | SumScore.java |
|  | |
| file | WindowAllScore.java |
|  | |
| file | WindowProcessor.java |
|  | |
| file | WindowScore.java |
|  | |
| file | WindowScoreIterator.java |
|  | |


---

Generated on Fri Aug 15 2014 15:11:16 for ESAT by  

 1.8.7
